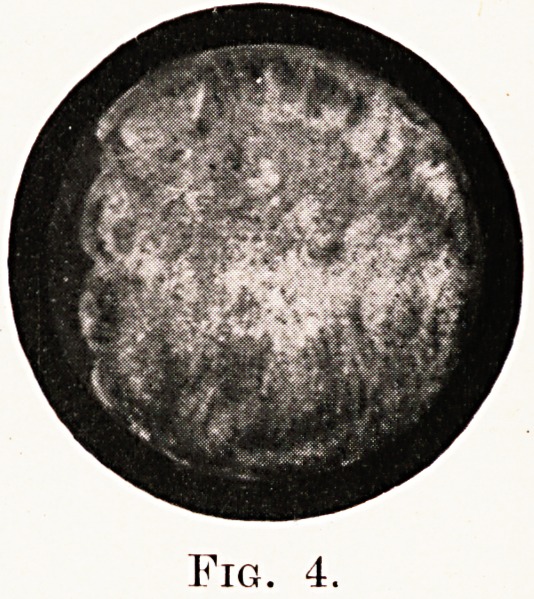# Bronchoscopic Diagnosis of Carcinoma of the Lung

**Published:** 1936

**Authors:** Gordon Scarff

**Affiliations:** Assistant Surgeon, Ear, Nose, and Throat Department, Bristol General Hospital


PLATE XIV

FIG. 1.

FIG. 3.

FIG. 2

FIG. 4.

### CARCINOMA OF THE BRONCHUS.

### Bronchoscopic Diagnosis of Carcinoma of the Lung.

By GORDON SCARFF, M.B., Ch.B., F.R.C.S.,
*Assistant Surgeon, Ear, Nose, and Throat Department, Bristol General Hospital.*

While the clinical and X-ray findings, which you have just heard, will make the diagnosis of carcinoma the most probable one, in order definitely to establish this diagnosis these cases are referred to the endoscopist. The air passages are examined directly by a bronchoscope for evidence of neoplasm, and a portion of the suspected tissue removed for microscopic examination.

On bronchoscopy the first noticeable feature is the fixation of the affected area of lung, which is in marked contrast to the normal range of mobility. There is usually some secondary infection present which may cause reddening of the mucosa, especially in the region of the growth. On reaching this area one finds the bronchus to present the appearances found in one of these drawings on the slide. In 9 cases examined 5 were right-sided and 4 were left, and for simplicity all the drawings are of the right bronchial tree. The first is copied from Chevalier Jackson's work, and shows the normal appearances on looking down the right bronchus. The second picture is the one most commonly seen—in 5 out of the 9 cases—where the affected bronchus is seen to be completely occluded, the lumen being indicated by a depressed area which may be central or lateral. In the first two cases examined this was taken to be due to pressure from without either by growth or an inflammatory mass, as the mucosa was intact and there was no sign of any ulceration, and no specimen was taken for biopsy, but in the later cases a small portion of mucosa was removed, and the submucous tissue was found to be infiltrated by growth of squamous or oat-celled type. The third picture shows diminution of the lumen of the lower lobe bronchus by a growth in the substance of the lung. A portion of the mucosa overlying this was reported on as doubtful, but the bronchoscopic appearance of the middle lobe bronchus was similar to the previous picture. He died from hæmoptysis four weeks later, and post-mortem examination showed scattered nodules throughout the lung substance. The fourth is from a man, aged 50, with very advanced clinical signs, in whom the right bronchus was almost completely blocked immediately below the bifurcation of the trachea by a mass of fungating growth.

The fixation, combined with any one of these bronchoscopic appearances, leaves little doubt as to the diagnosis from benign new growth and chronic inflammatory conditions, but a portion of the growth should always be taken for confirmation. Treatment by bronchoscopic means is limited to treatment by radium, and encouraging results are reported by others by means of implantation of a tube containing radon. As we have no radium emanation available, I have tried in two cases implantation of radium needles, but in each case these have been coughed out within twenty-four hours, so that some modification of this will have to be considered if this is to be an adjunct to treatment by other means.

## Figures and Tables

**Fig. 1. f1:**
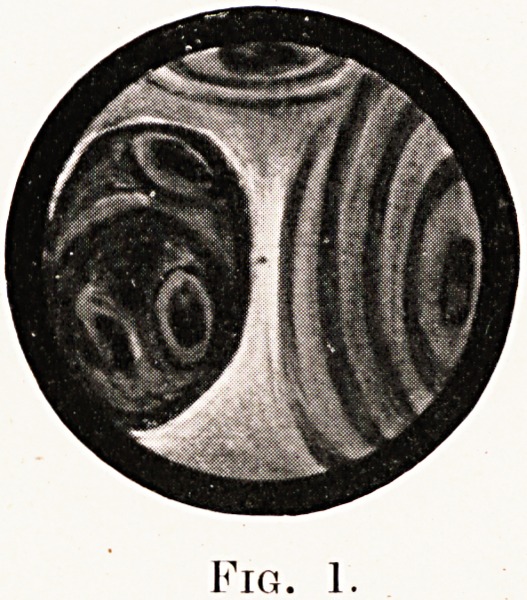


**Fig. 2 f2:**
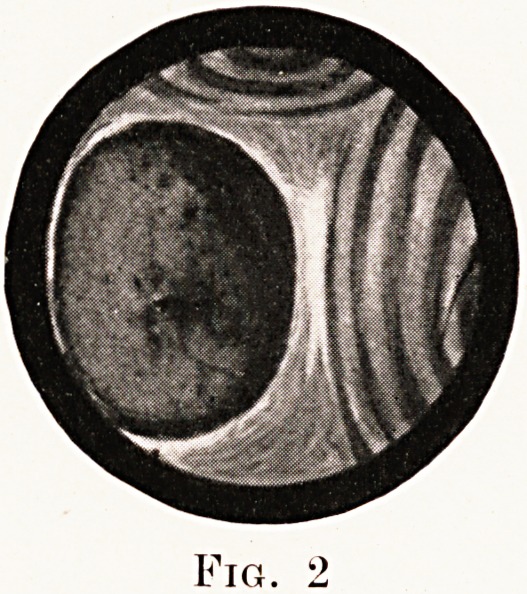


**Fig. 3. f3:**
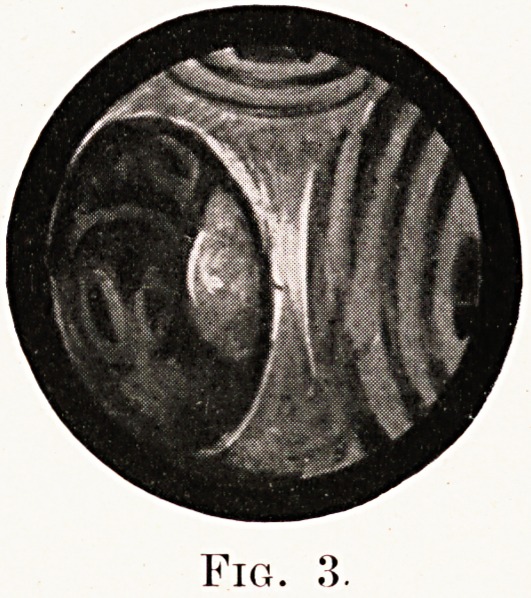


**Fig. 4. f4:**